# Delayed occurrence of traumatic aortic dissection? Biomechanical considerations and literature

**DOI:** 10.1007/s00414-022-02935-6

**Published:** 2022-12-17

**Authors:** H. Muggenthaler, D. Bismann, N. Eckardt, N. Gassler, M. Hubig, J. Shanmugam Subramaniam, G. Mall

**Affiliations:** 1 Institute of Legal Medicine, Jena University Hospital, Am Klinikum 1, 07747 Jena, Germany; 2 DEKRA Automobil GmbH, Auf dem Mittelfeld 3, 98693 Ilmenau Ilmenau, Germany; 3 Department of Radiology, Jena University Hospital, Am Klinikum 1, 07747 Jena, Germany; 4 Section Pathology, Institute of Legal Medicine, Jena University Hospital, Am Klinikum 1, 07747 Jena, Germany

**Keywords:** Aortic dissection, Aortic pseudoaneurysm, Motorcycle accident, Autopsy findings

## Abstract

Chronic aortic dissections and pseudoaneurysms caused by chest trauma are rare and generally have to be critically distinguished from non-traumatic dissections and aneurysms. We present a well-documented case of a post-traumatic aortic dissection that ruptured about 9 months after chest trauma. A motorcyclist sustained fractures of the forearm and chest trauma with paravertebral rib serial fractures and hemopneumothorax. Nine months after the accident, echocardiography revealed a pseudoaneurysm that ruptured 3 months later and 1 month prior to the planned surgery. An autopsy showed pericardial tamponade following a rupture of the dissected aorta. Accident scene documentation was consistent with a head-on collision of the motorcycle against the left front side of the car. The relative speed was about 55 km/h. Aggravation of unspecific symptoms after discharge, initial CT imaging, and the absence of atherosclerosis or medial necrosis hold for a post-traumatic genesis of the dissection in our case. Initially, the accident insurance company rejected the regulation. In the second instance, they revised rejection based on our interdisciplinary expert opinion.

## Introduction

Chronic aortic dissections and pseudoaneurysms caused by chest trauma are rare and generally have to be critically distinguished from non-traumatic dissections and aneurysms. There are just a few articles that deal with that issue, e.g. [1]. In traumatized patients, the authors in [2] found aortic injuries with an incidence of about 22 per 100,000 per annum. An incidence of 4.7 per 100,000 per annum of acute non-traumatic aortic dissections is given in [3]. There exists no statistical surveys for traumatic (chronic) aortic dissections.

There are different mechanisms causing aortic injuries, like relevant thorax compressions or high thorax accelerations [4]. Resultant tensile stresses can cause aortic lacerations or ruptures. Traumatic aortic dissections are most probably due to subliminal biomechanical loads not sufficient to cause ruptures or lacerations but to cause injuries of the intima with bleedings between the intima and the media. The dynamics of bleeding progression can be highly variable from several days up to several years [1]. Biomechanical experiments or thresholds are not available.

We present a well-documented case of a post-traumatic chronic aortic dissection that ruptured about 9 months after chest trauma. In the first instance, the accident insurance company rejected regulation for the reason that an idiopathic genesis was much more likely. Tasked with the preparation of an interdisciplinary expert opinion, we performed literature research and involved technical and different medical professions.

## Case

A 46-year-old man was riding his motorcycle (Kawasaki, LE650C), when suddenly the driver of a small passenger car (Hyundai i10) turned to the left and crossed the driveway of the motorcyclist. At the accident scene, the speed limit was 100 km/h. Although having initiated an emergency break, it came to a collision between the left front side of the car and the front wheel of the motorcycle. The motorcyclist was heard as a witness, and he estimated his pre-collision speed at about 60 km/h. He was thrown over the front hood of the car and came into his end position somewhere behind the point where the collision occurred. He further claimed that he was tumbling multiple times before approaching his final position. In the emergency room, he complained about chest and arm pain. During 9 months after the accident, his physical condition was getting worse, and an aortic aneurysm was detected in a transthoracic echo. Three months later and before planned surgery, the aneurysm ruptured.

## Autopsy findings and medical records

During in-patient emergency treatment after the accident, the following findings were found from diagnosis:

- Fractures of the right dorsal ribs 1–8, paravertebral
- Right small haemopneumothorax
- Retroperitoneal small contusion retroperitoneal right-sided
- Fractures of the right elbow (Monteggia), the right olecranon, the left radial head, the 1st and 2nd metacarpal bone and scaphoid bone of the left hand
- Initially high blood pressure, normalization during hospitalization

According to the medical records, there were no external injuries except a scrotal haematoma. The patient complained pain of the right hemithorax, the thoracic and lumbar spine, the renal bed and the upper extremities. There is no information regarding evacuated blood volume from the right pleural cavity. The rib fractures were treated conservatively. Polytrauma CT with contrast media did not show any signs of thoracic organ or vessel injury.

After discharge from hospital, the patient complained of unspecific discomforts like cephalgia, chronic fatigue and insomnia. About 9 months after the accident, he had to be treated for hypertension, and an echocardiography revealed a dilated ascending thoracic aorta, about 60 mm in diameter. The suspected diagnosis was an aortic aneurysm with an absolute indication of surgery. Surgery was planned; however, about 4 weeks prior to surgery, the non-hospitalized patient died. We could not evaluate the reason for not being in surgery soon after diagnosis.

The autopsy performed 12 months after accident and 3 months after aneurysm diagnosis revealed the following findings:

- Body length/mass: 1.84 m/90.4 kg
- Pericardial tamponade with 800 ml of blood
- Aortic laceration about 2 cm above the aortic valve
- Dissection involving the whole thoracic aorta from the diaphragmatic passage to the heart
- Increased heart mass (590 g) and a 2-cm-thick wall of the left ventricle
- No signs of arteriosclerosis of the aortic and carotid artery walls

The cause of death was a pericardial tamponade following a rupture of the dissected aorta. Histopathological analysis did not reveal any pathological findings of the vessels or the heart.

**Fig. 1:**
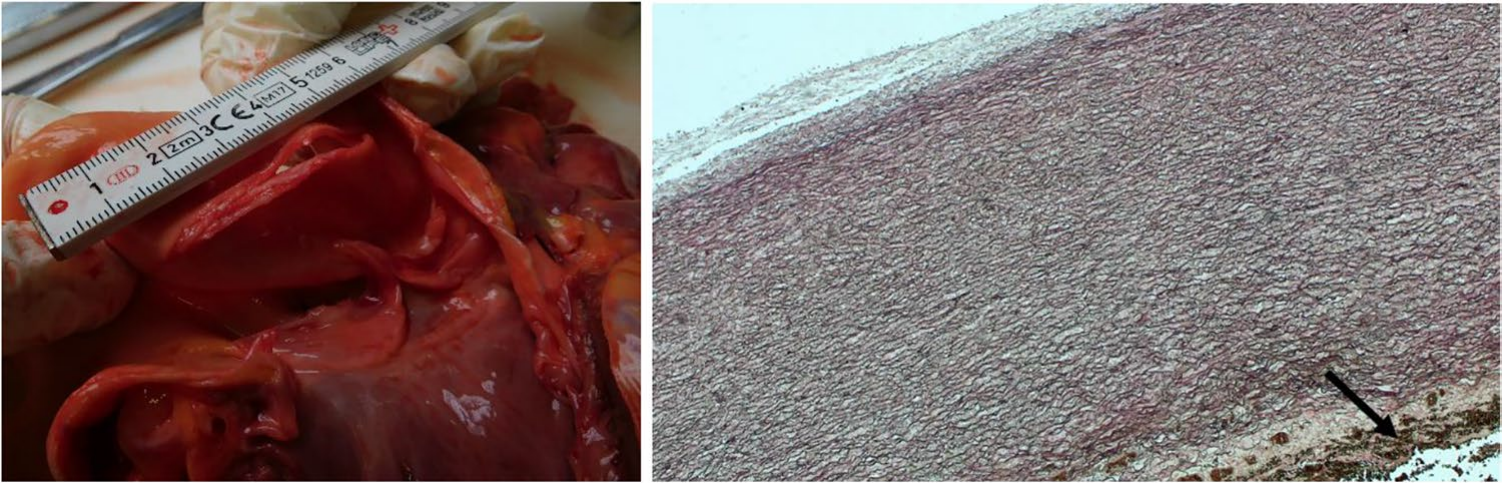
Aortic laceration in the autopsy (left) and histological section of the aorta, 50 × magnification (right)

Figure 1 shows the aortic rupture as seen in the autopsy as well as a histological section of the aorta (EvG staining) with intact elastic fibres and without mucoid degeneration. There were signs of bleeding in the adventitia caused by trauma and dissection, respectively. The rupture was about 3.5 cm in length. With respect to the aortic longitudinal axis, the rupture had an oblique orientation.

**Fig. 2:**
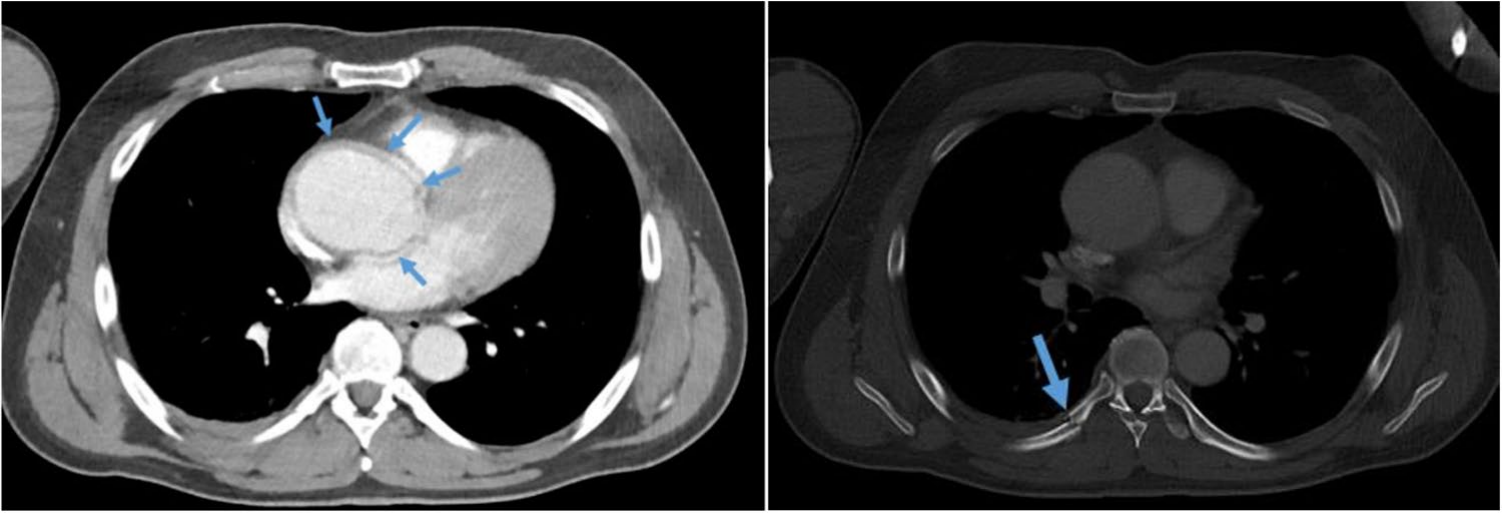
Polytrauma CT after the accident. Left, axial reconstruction in soft tissue window with sickle-shaped contrast signal surrounding the ascending aorta. Right, axial reconstruction in bone window with paravertebral rib fracture of the right 7^th^ rib

In preparation of the expert opinion, a radiologist reviewed the polytrauma CT performed after the motorcycle accident. Besides the findings documented in the medical report, he found a sickle-shaped contrast signal surrounding the ascending aorta indicating an aortic wall haematoma (Fig. 2, left). However, this finding could also be an artefact caused by cardiac action with recurring increase of aortic lumen. The fracture of the right 7th rib is shown in Fig. 2 on the right-hand side. All the injured ribs fractured near the distal end of the transverse process.

## Accident and injury reconstruction

**Fig. 3:**
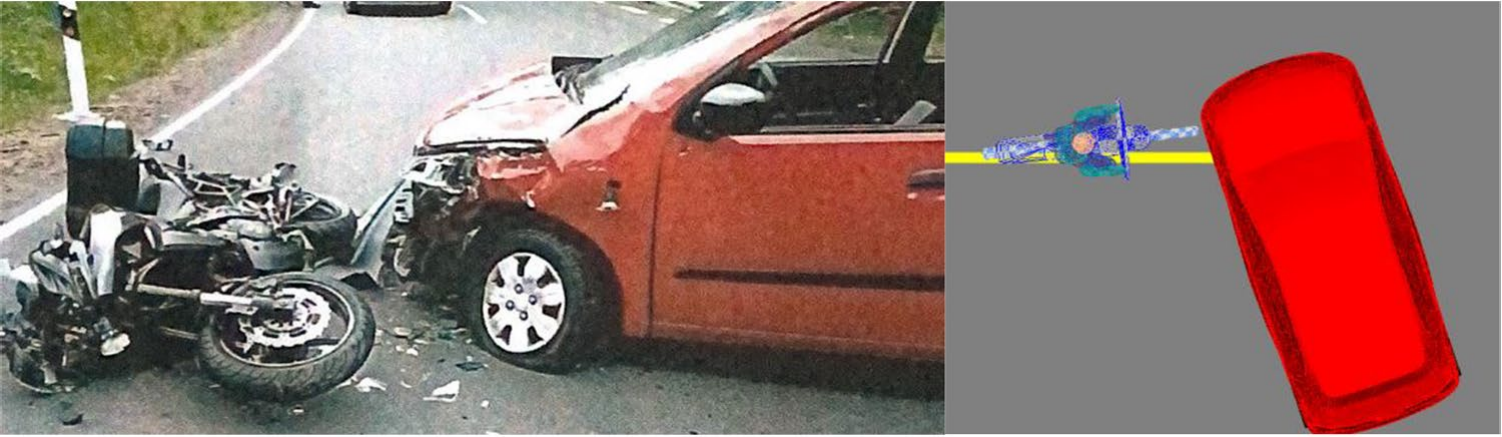
End positions of the vehicles (left), collision position in PC-Crash (right)

Unfortunately, there was no detailed documentation of the accident scene depicting the exact end positions of the motorcyclist. Photos taken by the police show the motorcycle lying on the right side in front of the car, indicating a rotation around its vertical axis of around 180° due to the collision. The car shows considerable damage to the left front corner and some deformations of the left A-pillar (Fig. 3, left).

Based on the photographs and on the deformations of the vehicles, the probable collision position was reconstructed using the accident reconstruction software PC-Crash. Plausible kinematics of both vehicles were determined by pre-impact speeds of 55 km/h for the motorcycle model and 8 km/h for the car model. From a technical point of view, the damages to the vehicles correspond to the relative speed assumed in the reconstruction software. Because of insufficient scene documentation, a more reliable reconstruction of the accident was not possible.

**Fig. 4:**
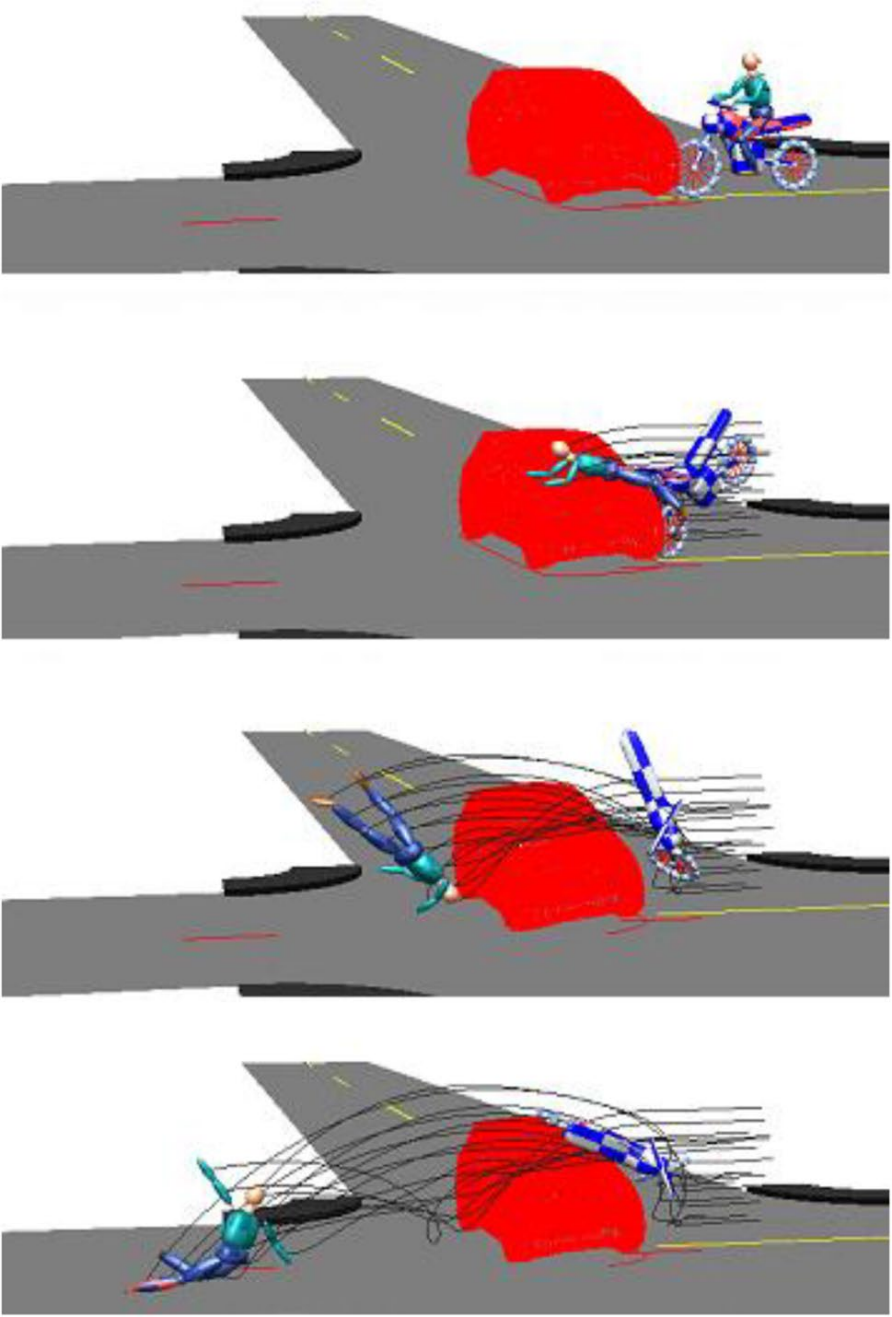
Kinematics of the motorcyclist

Figure 4 depicts the kinematics of the motorcyclist model with a trajectory over the front hood of the car. In the simulation, the model lands with its back on the roadway. The thoracic injuries most probably occurred during a secondary impact on the road, while the fractures of the upper extremities are typical injuries due to interactions with the handlebar, the car etc. The kinematics also suggests a potential abdominal contact with the PTW handlebar. However, paravertebral rib fractures as well as the retroperitoneal contusion rather can be explained by a dorsal impact than by a handlebar interaction. We refrain from an analysis of contact forces and impact velocities, because in our opinion, the collision is too complex to be evaluated using simple multibody models and without detailed scene documentation.

## Discussion

The autopsy revealed a pericardial tamponade due to a ruptured dissected aorta. Morphologically, a differentiation in terms of post-traumatic or pathologic/idiopathic genesis was not possible.

High chest accelerations as well as thoracic deformations [5] can cause high tensile stresses especially in the aortic arch with severe injury like lacerations. We assume a very similar mechanism for traumatic dissections with tensile stresses causing failure of the intima followed by an accumulation of blood between the intima and the media. However, biomechanical tolerance values concerning traumatic aortic dissections do not exist. According to Otte et al. [6], the cumulative probability of aortic injuries in terms of lacerations and ruptures significantly increased in motorcycle — car accidents at relative speeds higher than 50 km/h. In tests with PMHS (post-mortem human subject), Cavanaugh et al. found aortic injuries in side impacts with impact speeds between 24 and 37 km/h [7]. The accident in our case with an impact speed of about 55 km/h seemed to be adequate to cause injuries to the intima with subsequent formation of a dissection. A quantitative biomechanical reconstruction of the thoracic load based on the simulation in Fig. 4 is not possible. A potentially high trajectory of the motorcyclist in combination with a relevant rotational component could lead to relevant impact energies during ground contact. The injuries sustained in the motorcycle accident proved blunt impact force against the laterodorsal chest, indicating considerable thoracic acceleration.

Comparable cases with delayed occurrence of a traumatic aortic dissection can be found in literature. Beslic et al. [1] describe eight cases with post-traumatic aortic dissections diagnosed between 7 days and 18 years after the accident. In [8], eight cases with post-traumatic pseudoaneurysms are reported with surgical treatment between 8 weeks and 18 years after primary injury. After a head-on collision, a 20-year-old driver complained about left chest pain with minor bruises to the chest wall. The x-ray examination revealed a haemothorax without any fractures. About 2 years later, a post-traumatic aneurysm was detected [9]. Bizzarri et al. [10] and Hirose and Svensson [11] also report cases with chronic post-traumatic aortic aneurysms.

The polytrauma scan during primary care in our case showed a sickle-shaped contrast signal surrounding the ascending aorta, which retrospectively can be interpreted as a sign of acute aortic injury, though without giving evidence of acute aortic injury. In the emergency department, high blood pressure values were documented. Hypertension can either be a risk factor for idiopathic aortic dissections or a symptom of acute aortic injury [12]. Left ventricular wall thickening of about 2 cm was the only correlate in post-mortem investigation that raised suspicion of the presence of systematic disease, like hypertension. Histopathologic screening showed neither progressed atherosclerosis nor medial necrosis. In the absence of medial necrosis, hypertension as the reason for the dissection seemed unlikely.

In summary, the absence of medial necrosis in histopathologic screening, the sickle-shaped contrast signal surrounding the ascending aorta in CT imaging and the chest trauma 12 months before aortic rupture hold for a post-traumatic genesis of the dissection in our case. Moreover, the aggravation of unspecific symptoms between discharge and diagnosis of the pseudoaneurysm support the post-traumatic origin hypothesis of the dissection.

Based on our interdisciplinary expert opinion, the accident insurance company admitted injury causality.

## Data Availability

Not applicable.
